# The ERA-Related GTPase AtERG2 Associated with Mitochondria 18S RNA Is Essential for Early Embryo Development in *Arabidopsis*

**DOI:** 10.3389/fpls.2018.00182

**Published:** 2018-02-15

**Authors:** Pengyu Cheng, Hongjuan Li, Linlin Yuan, Huiyong Li, Lele Xi, Junjie Zhang, Jin Liu, Yingdian Wang, Heping Zhao, Huixin Zhao, Shengcheng Han

**Affiliations:** ^1^Beijing Key Laboratory of Gene Resource and Molecular Development, College of Life Sciences, Beijing Normal University, Beijing, China; ^2^Xinjiang Key Laboratory of Special Species Conservation and Regulatory Biology, College of Life Science, Xinjiang Normal University, Urumqi, China; ^3^Key Laboratory of Cell Proliferation and Regulation Biology, Ministry of Education, College of Life Sciences, Beijing Normal University, Beijing, China

**Keywords:** AtERG2, mitochondria 18S RNA, early embryo development, ROS, cell death, *Arabidopsis*

## Abstract

The ERA (*E. coli* RAS-like protein)-related GTPase (ERG) is a nuclear-encoded GTPase with two conserved domains: a GTPase domain and a K Homology (KH) domain. ERG plays a vital role in early seed development in *Antirrhinum majus*. However, the mechanism that regulates seed development remains unclear. Blasting the genome sequence revealed two homologies of ERG, AtERG1, and AtERG2 in *Arabidopsis*. In this study, we found that AtERG2 is localized in the mitochondria and binds mitochondrial 18S RNA. Promoter and transcript analyses indicated that *AtERG2* was mainly expressed in the leaf vein, trichome, and ovule. The T-DNA insertion lines of *AtERG2* showed silique shortage, early seed abortion, and sporophytic maternal effects (SME), in which some seeds arrested in the zygotic stage at 1.5 days after pollination (DAP) and aborted at 2.0 DAP in *aterg2-1* +*/*−. We further showed that the ovules of these arrested seeds presented unusual tissue degradation inside the embryo sacs. Reactive oxygen species (ROS) accumulated at 1.0 and 1.5 DAP in the arrested seeds, and the transcription of several ROS-responsive genes, *WRKY40, ANAC017*, and *AOX1a*, was up-regulated in the *aterg2-1* +*/*− arrested seeds at 1.5 and 2.0 DAP, but not in wild-type (WT) and *aterg2-1* +*/*− developed seeds. The cell death-related gene *BAG6* was also transcriptionally activated in *aterg2-1* +*/*− seeds arrested at 2.0 DAP. Additionally, the protein level of mitochondria protein ATPase Subunit 6 was lower in 2-DAP siliques of *aterg2-1* +*/*− than it was in those of WT. These results suggested that AtERG2 promotes early seed development by affecting the maturation of the mitochondria ribosome small subunit and mitochondrial protein translation in *Arabidopsis*.

## Introduction

The small GTP-binding proteins (GTPases) are found in all domains of life and act as molecular switches that are “activated” by GTP and “inactivated” by the hydrolysis of GTP to GDP to regulate numerous cellular processes, such as signal transduction, reorganization of the cellular cytoskeleton, regulation of transcription and translation, protein transport and vesicle trafficking (Takai et al., 2001; Vernoud et al., 2003; Shan, 2016). Based on the sequences and structural signatures, the small GTPase superclass can be divided into two large classes: the translation factors (TRAFAC) class and the signal recognition particle (SRP), MinD-like ATPases, and BioD (SIMIBI) class. The TRAFAC class includes proteins involved in translation, signal transduction (in particular, the extended Ras-like family), and cell motility. The SIMIBI class is involved in protein localization, chromosome partitioning, membrane transport, and a group of metabolic enzymes with kinase or related phosphate transferase activity (Leipe et al., 2002). In 1986, a yeast Ras-like protein that contains 316 amino acids and low GTP hydrolyse activity was found in *E. coli* and named *E. coli* Ras-like protein (ERA) (Ahnn et al., 1986). ERA is essential for cell growth and viability (Inada et al., 1989), cell division (Britton et al., 1997), and pleiotropic processes, including carbon metabolism, fatty acid metabolism, and adaptation to thermal stress (Lerner and Inouye, 1991; Voshol et al., 2015). ERA has two conserved domains: the GTPase domain, which is homologous to RAS and belongs to the TRAFAC class, and the K Homology (KH) domain, which binds to the *E. coli* 16S rRNA and the 30S ribosomal subunits (Meier et al., 1999). Tu et al. showed that the GTP-binding form of ERA is helpful for recognition and binding to the (1530)GAUCACCUCC(1539) sequence at the 3′ end of 16S rRNA, and RNA recognition stimulates its GTP-hydrolysing activity and the switch to the GDP-binding form; the latter suggested that ERA acts as a chaperone for the processing and maturation of 16S rRNA to benefit the assembly of the 30S ribosomal subunit (Tu et al., 2009, 2011).

ERA is highly conserved among bacteria, archaea, and eukaryotes, and its homolog in humans is called Era-like 1 (ERAL1), which is nuclear-encoded and localized in the mitochondria matrix (Britton et al., 2000; Uchiumi et al., 2010). Previous studies showed that ERAL1 binds mitochondria 12S rRNA, but not cytoplasmic ribosome rRNA, to help the maturation of the mitochondria small ribosomal subunit, which is similar to Era (Dennerlein et al., 2010; Uchiumi et al., 2010; Uchiumi and Kang, 2012). Prior studies showed that knockdown of ERAL1 by siRNA inhibits mitochondrial protein translation and elevates mitochondrial reactive oxygen species (ROS) production, leading to cell death (Uchiumi et al., 2010; Xie et al., 2012). These results indicated that ERAL1 plays an important role in the small ribosomal constitution and is involved in cell viability.

In plants, *ERA-related GTPase* (*ERG*) was first found in *Antirrhinum majus* and is expressed in dividing or metabolically active cells. ERG was predicted to target to the mitochondria by amino-terminal sequences, and a deletion allele of *ERG* by site-selected transposon mutagenesis resulted in the arrested development of seeds containing embryos and endosperm after fertilization (Ingram et al., 1998). Genomic data on the *ERG* genes in *Arabidopsis* showed that there are two homologs, AtERG1, localized in chloroplast, and AtERG2, localized in the mitochondria (Suwastika et al., 2014). Jeon et al (Jeon et al., 2014) showed that *Nicotiana benthamiana* double Era-like GTPase (NbDER) contains two tandemly repeated GTPase domains and a C-terminal KH-like domain involved in RNA binding. NbDER was localized primarily to chloroplast nucleoids, possessed GTPase activity, and bound to 23S and 16S ribosomal RNAs, which contributed to the maturation and assembly of the chloroplast 50S ribosomal subunit. Depletion of NbDER impaired processing of plastid-encoded ribosomal RNAs, resulting in accumulation of the precursor rRNAs in the chloroplasts and a leaf-yellowing phenotype caused by disrupted chloroplast biogenesis. However, the mechanism of AtERGs involved in the organelle rRNA processing and ribosome biogenesis remains unclear. Here, we showed that AtERG2 was localized in the mitochondria, dependent on its N-terminal sequence, and bound mitochondria 18S RNA *in vivo*. The T-DNA insertion lines of *ATERG2* severely affect the seed development, and some seeds arrest at the zygote stage at 1.5 DAP and abort at 2.0 DAP in *aterg2-1* +*/*−. We further found that ROS were accumulated; the transcription of several ROS-responsible genes, *WRKY40, ANAC017*, and *AOX1a*, and the cell death-related gene, *BAG6*, were up-regulated in the *aterg2-1* +*/*− arrested seeds, but not in WT and *aterg2-1* +*/*− developed seeds, and the protein level of ATPase S6 was lower in the *aterg2-1* +*/*− siliques at 2 DAP than it was in those of WT. These results suggest that AtERG2 is involved in regulating seed early development through mitochondria ribosome maturation and protein translation.

## Materials and methods

### Plant growth

*Arabidopsis thaliana* (Columbia-0) plants were grown in soil with a 16/8-h light/dark cycle at 22°C in the plant growth room. Seeds were sterilized and plated onto 1/2 Murashige and Skoog (MS) basal salt medium with 0.8% agar and 1.5% (w/v) sucrose in the dark for 2 days at 4°C. Then, the plates were grown in the growth chamber MLR-352H-PC (Panasonic, Japan) at a 16/8-h light/dark cycle at 22°C for 7 days, and the seedlings were transplanted in the soil for continual growth. The siliques were collected at the exact developmental stages for the next analysis.

### DNA and RNA extraction

*Arabidopsis* genomic DNA was isolated from 4-week-old leaves using the extraction buffer [200 mM Tris-HCl pH 7.5, 250 mM NaCl, 25 mM EDTA, 0.5% sodium dodecyl sulfate (SDS)] as described (Edwards et al., 1991). Total RNA was extracted by TRIZOL Reagent (Thermo Fisher Scientific, USA) from *Arabidopsis* seeds according to the manufacturer's instructions (Thermo Fisher Scientific) and quantified using a Nanodrop ND-1000 spectrophotometer (LabTech, USA). Total RNA (5 μg) was used for cDNA synthesis with Superscript II RNase H-Reverse Transcriptase (Life Technologies, USA) and oligo(dT)_18_ primers according to the manufacturer's instructions.

### Characterization of T-DNA insertion lines of *AtERG2*

Two T-DNA insertion lines of *AtERG2: aterg2-1* (SALK_032115) and *aterg2-2* (SALK_032124) were purchased from *Arabidopsis* stock centers (http://www.arabidopsis.org/). The genotype of T-DNA lines was confirmed by PCR using the gene-specific primers and a T-DNA–specific primer (Table S1).

### Constructs and transgenic plants

The coding sequence of *AtERG2* was amplified by PCR and cloned into the Gateway Cloning vector pE6c or pE3c (Addgene, USA) with Bam HI and Not I sites. To generate the various truncated AtERG2 constructs, such as AtERG2_N (containing only N-terminal), AtERG2_C [containing GTPase domain (GD) and KH domain (KH)], AtERG2_GD (containing only GD), and AtERG2_KH (containing only KH), the corresponding AtERG2 fragments were amplified and subcloned into pE6c. Construct maps containing different parts of AtERG2 are shown in Figure S2. The different constructs were used in combination with the destination vector pMDC32 (http://www.arabidopsis.org/) with the Gateway LR II kit (Life Technologies, USA) to generate the plant expression vectors. A genomic fragment of 1.8 kb in the promoter region of *AtERG2* was amplified by PCR and cloned into a GUS binary vector pCAMBIA 1305.1 with EcoR I and Bgl II sites. The primers used in these constructs are listed in Table S1. All vectors were confirmed by sequencing.

The resulting *35S::*AtERG2-6XMyc and *P*_*AtERG*2_*::*GUS constructs were transformed into *Agrobacterium tumefaciens* GV3101. Transgenic plants were generated by the floral dip method (Clough and Bent, 1998) and selected on MS medium containing hygromycin B (25 mg/L) or kanamycin (50 mg/L). More than 100 AtERG2-6XMyc or *P*_*AtERG*2_*::*GUS lines were recovered. Independent homozygous transformants carrying a single insertion in the T3 generation were further analyzed.

### Transient transformation of *Arabidopsis* mesophyll protoplasts

The vectors containing AtERG2-eYFPs were co-transformed with a mCherry-tagged organelle marker gene and transiently expressed in *Arabidopsis* mesophyll protoplasts, as described previously (Liang et al., 2015). After incubation for 16 h at 22°C, fluorescence was visualized using an LSM 700 confocal microscope (Zeiss, Germany). Observations were made using a 63 × objective under oil immersion. eYFP fluorescence was excited at 488 nm and collected at SP 550 IR. The mCherry fluorescence was stimulated at 555 nm and collected at SP 630 IR.

### Immunoprecipitation of mitochondria 18S RNA with AtERG2

The 2-week-old transgenic *Arabidopsis* seedlings overexpressing AtERG2-6Xmyc were ground in liquid nitrogen, and the protein/RNA complexes were extracted using equal volumes of lysis buffer (IP buffer (50 mM NaCl, 2 mM MgCl_2_, 10 mM HEPES pH 7.5, 2 mM DTT, 2 mM EDTA, 100 U/mL RNasin RNase inhibitor [Promega, USA], and 100 U/mL protease inhibitor cocktail [Sigma-Aldrich, USA] plus 1% TritonX-100 and 0.5% SDS) and incubated for 1 h on ice, then centrifuged twice at 13,000 g for 10 min at 4°C to remove the insoluble debris. The supernatant was diluted with nine volumes of IP Buffer and incubated with 10 μL anti-Myc antibody (Sigma-Aldrich) for 4 h on ice with occasional gentle mixing. Next, the anti-Myc-decorated extracts were incubated with 10 μl protein G agarose beads pre-washed with IP buffer for 1 h at 4°C with rotation. Then, the beads were spun down at 10,000 rpm in a microfuge for 1 min at 4°C, and washed eight times with 1 mL washing buffer (100 mM NaCl, 2 mM MgCl_2_, 10 mM HEPES pH 7.5, 2 mM DTT, 2 mM EDTA, 100 U/mL RNasin RNase inhibitor, and 100 U/mL protease inhibitor cocktail). After elution with TRIZOL reagent, co-immunoprecipitated RNA was isolated and analyzed by quantitative real-time RT-PCR (qRT-PCR).

### qRT-PCR

qRT-PCR was performed on the 7500 Fast Real-Time PCR System (Applied Biosystems, USA) using Power SYBR® Green PCR Master Mix (Applied Biosystems). The thermal program was 2 min at 50°C, 10 min at 95°C, followed by 40 cycles of 15 s at 95°C and 60 s at 60°C. The data were normalized to the expression of *Arabidopsis actin*. The dissociation curve program was used to confirm the specificity of the target amplification product. All primers used in this study are listed in Table S1. At least three independent biological replicates were performed for qRT-PCR analysis.

### Microscopy analysis

Seeds were removed from siliques at different developmental stages, cleared in Hoyers solution (7.5 g gum arabic, 100 g chloral hydrate, 5 mL glycerol in 30 mL water) and examined using a Zeiss Axio Observer A1 microscope equipped with DIC optics as described by Liu and Meinke (1998). The ROS detection was performed according to a modified version of a previously described method (Daudi et al., 2012) by staining with 1 mg/mL 3,3′-diaminobenzidine (DAB, Sigma) containing Triton X-100 (0.1% v/v) and 10 mM sodium phosphate buffer (pH 7.0). As previously described (Yu et al., 2005), the GUS staining was performed for 4 h with seedlings and leaves or overnight with flowers and seeds at 37°C in GUS staining solution (50 mM sodium phosphate buffer, pH 7.0, 10 mM EDTA, 0.1% Triton X-100, 2 mM potassium ferricyanide, 2 mM potassium ferrocyanide, 1 mg/mL X-Gluc) before the clearing procedure.

### Whole-mount Eosin B staining-confocal laser scanning microscopy (WE-CLSM) analysis

The seeds from 1.5- and 2.0-DAP siliques were harvested and fixed overnight in FAA solution (90 mL 50% acetic acid; 5 mL glycerine; 5 mL 37% formaldehyde). After gradual rehydration (100, 90, 70, 50, 30, and 10% ethanol) for 20 mins, the materials were stained twice in 10 mg/mL Eosin B (2 h each). Subsequently, the sample was gradually dehydrated (10, 30, 50, 70, 90, and 100% ethanol) for 20 mins until the solvent was replaced by 100% ethanol. Finally, seeds were cleared with methyl salicylate and observed using an LSM 700 confocal microscope (Zeiss, Germany).

### Western blot assays

The total proteins were extracted from 2.0-DAP siliques in extraction buffer (50 mM Tris-HCl [PH 8.0], 200 mM NaCl, 1 mM β-mercaptoethanol, 2% SDS, 10% glycerin, and 0.5% Triton-X 100), and the protein concentration was determined using a Bio-Rad Protein Assay Kit with bovine serum albumin (BSA) as the standard. The samples were separated by SDS-polyacrylamide gel electrophoresis (SDS-PAGE) and transferred to a polyvinylidene difluoride (PVDF) membrane (Millipore, USA) under a 200-mA constant current for 45 min in transfer buffer (12.5 mM Tris-HCl, 192 mM glycine, and 10% methanol; pH 8.3). Anti-ATPase Subunit 6 (1:1,000, Thermo Fisher Scientific) and anti-actin (1:1,000, ZSGB-BIO, China) antibodies were used as the primary antibodies, and horseradish peroxidase-conjugated goat anti-rabbit IgG antibodies (1:2,000, Cell Signaling Technology, USA) were used as the secondary antibodies. Signals were detected using the automatic chemiluminescence image analysis system Tanon 5200 Multi (Tanon, China).

### Statistical analysis

All statistical analyses were performed using the Data Processing System (Tang and Zhang, 2013). One-way analysis of variance (ANOVA) and Tukey's multiple range test were conducted to determine statistical significance. In the study, the letters a–e or ^*^ indicate statistical significance (*P* < 0.05), and ^***^ indicates high levels of statistical significance (*P* < 0.001).

## Results

### Phylogenetic relationship of AtERGs and its homologs

A previous study showed that there are two homologs of ERG in the *Arabidopsis* genome: AtERG1 (At5g66470), localized in the chloroplast, and AtERG2 (At1g30960), localized in the mitochondria (Suwastika et al., 2014). We used the sequence of ERA, ERAL1, ERG, AtERG1, and AtERG2 to search NCBI public databases (http://blast.ncbi.nlm.nih.gov) at an *E*-value of 1e-10. The presence of conserved GTPase and KH domains is the exclusive criterion for confirmation of 16 ERA homologous proteins in 14 species, which include MmERA from *Mus musculus*, DrERA from *Danio rerio*, DmERA from *Drosophila melanogaster*; ScERA from *Saccharomyces cerevisiae*, OsERG1 and OsERG2 from *Oryza sativa*, CsERG from *Cucumis sativus*, ZmERG from *Zea mays*, NtERG from *Nicotiana tabacum*, StERG from *Solanum tuberosum*, and SlERG from *Solanum lycopersicum*. Additionally, Jeon et al. (2014) found a chloroplast-localized double Era-like GTPase in *Nicotiana benthamiana* (NbDER), which binds to chloroplast 23S and 16S ribosomal RNAs. We further identified the homologs of NbDER, such as AtDER from *Arabidopsis thaliana*, EcDER from *E. coli*, ZmDER from *Zea mays*, and OsDER from *Oryza sativa*. Then, a phylogenetic tree was generated using the CDS (sequence coding for amino acids in protein) of 21 ERA homologs, and we found that members of the ERA homologs were separated into four distinct clades, designated I, II, III, and IV (Figure S1). Clade I included 12 members: ERG, AtERG2, CsERG, OsERG2, ZmERG, NtERG, SIERG, StERG, ERAL1, MmERAL1, DrERAL1, and DmERAL1, which were predicted and/or identified as mitochondria-localized. Clade II contained three members: ERA, AtERG1, and OsERG1; this suggested that ERA is most closely related to chloroplast-localized AtERG1 and OsERG1. Clade III included one member: ScERA. Clade IV had five DERs. These eukaryotic ERALs and ERGs were nuclear-encoded and organelle-localized genes, indicating that they were transferred to the nucleus after organelle symbiogenesis.

### AtERG2 is dependent on its N-terminal sequence for localization to the mitochondria

**Figure 1:**
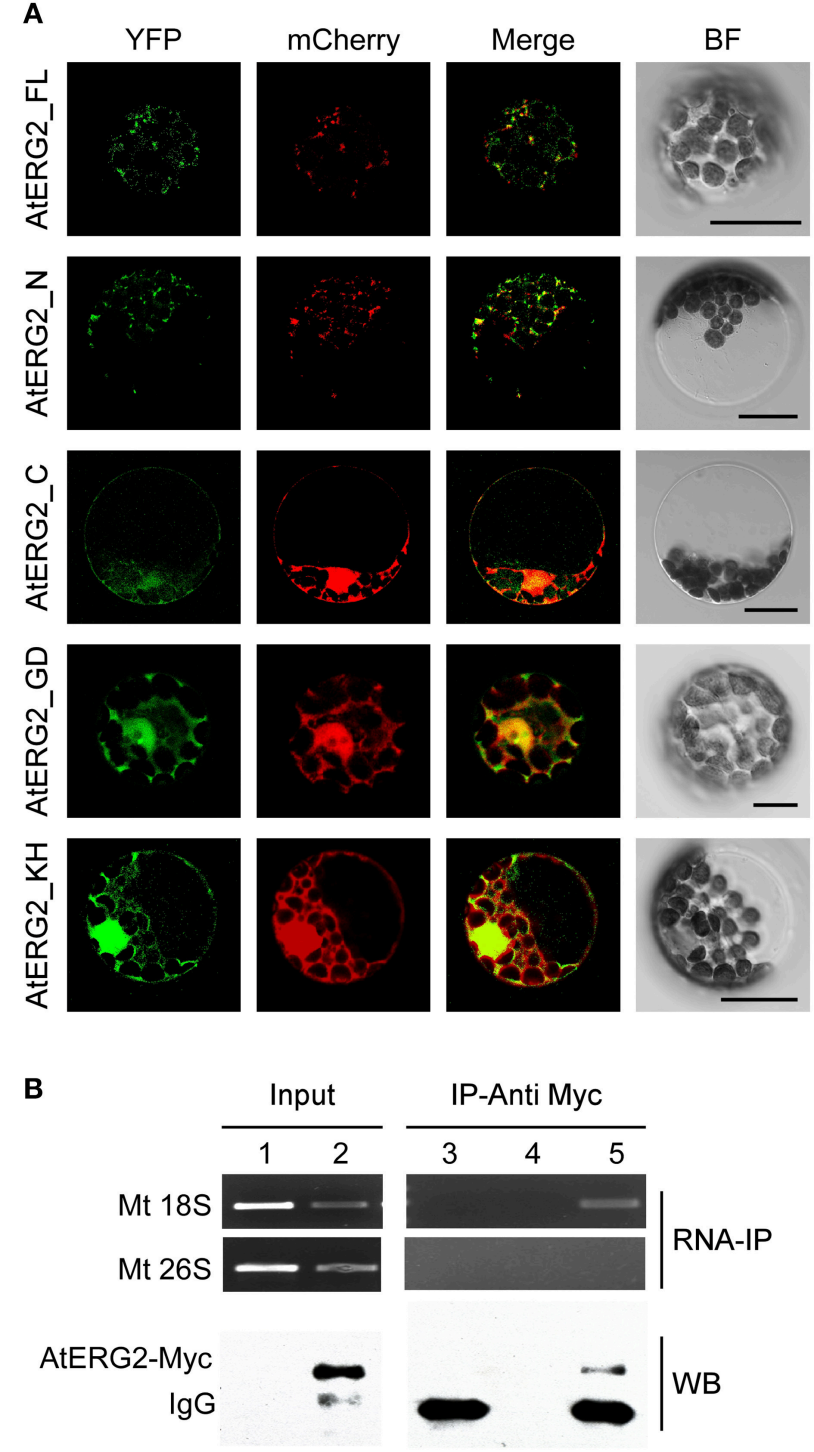
AtERG2 is localized in mitochondria and associated with mitochondria 18S RNA. **(A)** Localization of various truncated AtERG2 constructs in *Arabidopsis* mesophyll cells. The full-length AtERG2 (AtERG2_FL-YFP) and AtERG2 N-terminal part (AtERG2_N-YFP) were co-transformed with mCherry-labeled mitochondria marker; AtERG2 C-part (AtERG2_C-YFP), AtERG2 GTPase domain (AtERG2_GD-YFP), and AtERG2 KH domain (AtERG2_KH-YFP) co-transformed with mCherry-labeled cytoplasm marker. All images in this figure were obtained from one optic section. Scale bars are equivalent to 20 μm. The schematic of different AtERG2-eYFP constructs were shown in Figure S3. **(B)** RNA immunoprecipitation (RNA-IP) assays (top) showing the interaction between AtERG2-MYC and Mt 18S RNA in *Arabidopsis* were performed with (Lane 3 and Lane 5) or without (Lane 4) MYC antibody followed by RT-PCR detection. Mt 28S RNA served as a negative control (Lane 1 and Lane 2). The Western blot (WB, bottom) indicates the protein level in the transgenic plants, which was detected prior to the RNA-IP assay.

To further investigate the subcellular localization of AtERG2, we developed various constructs containing different parts of AtERG2 fused with yellow fluorescent protein (eYFP) at their C-terminal end (Figure S2) and transiently co-transformed cells with a mCherry-labeled organelle marker in the *Arabidopsis* mesophyll protoplasts. The full-length AtERG2 was localized in the mitochondria as previously reported (Figure 1A; Suwastika et al., 2014). We further found that the N-terminal end (1–150 aa) of AtERG2 was localized in the mitochondria. However, the C-terminal end, GTPase domain, and KH domain alone diffused into the cytoplasm (Figure 1A). These results indicated that the N-terminal end of AtERG2 is critical for its localization to the mitochondria.

### AtERG2 associates with mitochondria 18S rRNA *in Vivo*

We next used RNA immunoprecipitation (RNA-IP) assays to detect AtERG2 binding ribosome rRNA. The AtERG2-Myc:rRNA complex was extracted from the transgenic *Arabidopsis* stably expressed AtERG2-Myc fusion protein, preincubated with Myc antibody, and purified by protein G agarose beads. Then, the rRNA was isolated with Trizol solution and detected by RT-PCR (Figure 1B). The results showed that AtERG2 binds mitochondria ribosome small subunit 18S rRNA but not large subunit component 26S rRNA, suggesting that AtERG2 plays the same role as its homologs ERA and ERAL1 for the maturation of 16S rRNA and assembly of ribosomes.

### *AtERG2* is mainly expressed in the leaf vein, trichome, mature pollen, and ovule

**Figure 2:**
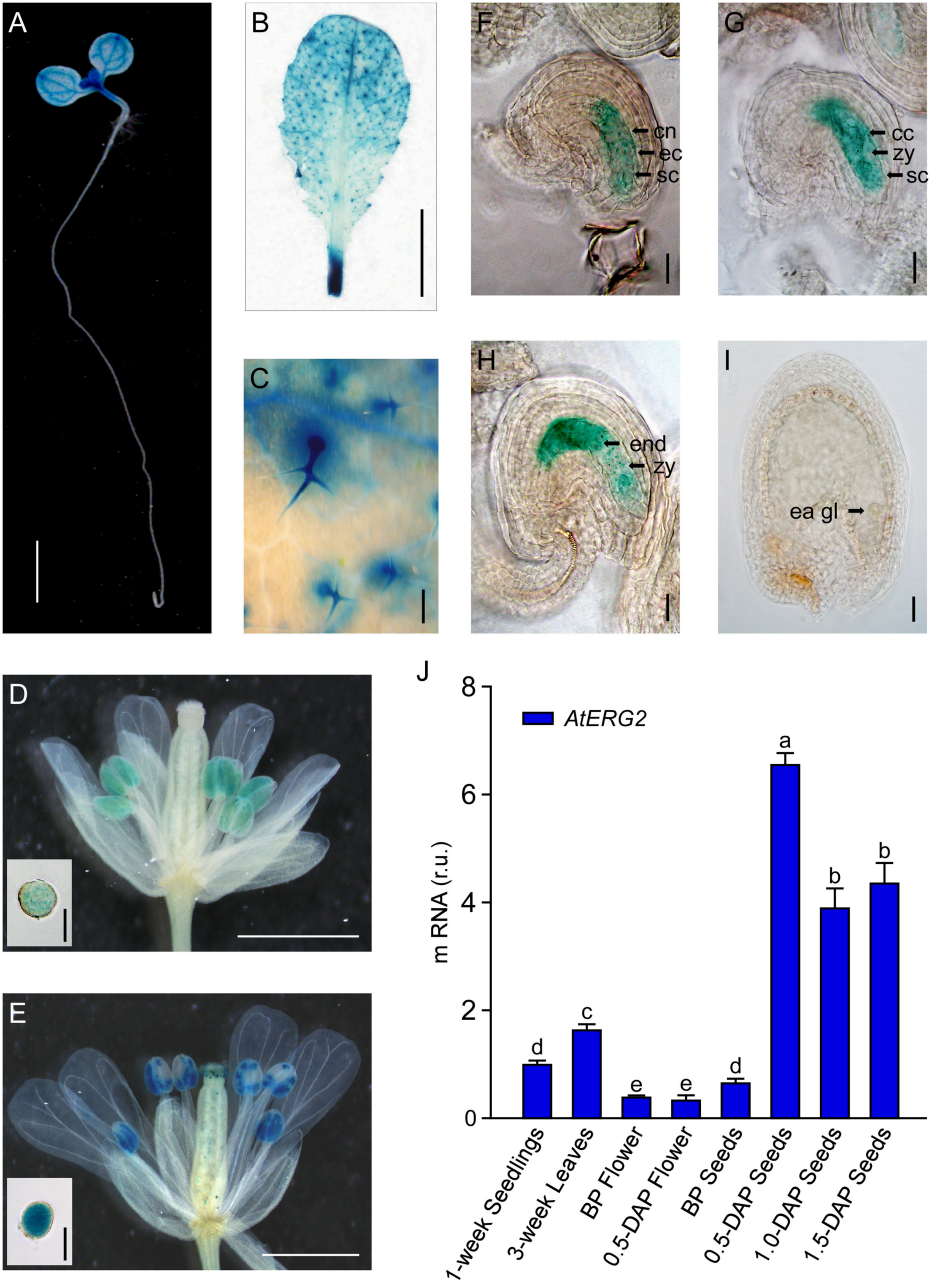
Differential Expression of *AtERG2* in pollen and early-stage ovule. GUS staining of *P*_*AtERG*2_::GUS transgenic *Arabidopsis* plants for 4 h with seedlings and leaves or overnight with flowers and seeds. Bar = 2 mm in **(A)**; 5 mm in **(B)**; 0.2 mm in **(C)**; 0.5 mm in **(D,E)**; 0.05 mm in **(F–I)**; and 0.02 mm in pollen of **(D,E)**. **(A)** *P*_*AtERG*2_::GUS fusion staining in 1-week-old *Arabidopsis* seedling showing GUS activity in cotyledon and shoot apical meristem. **(B,C)** *P*_*AtERG*2_::GUS fusion staining in 4-week-old *Arabidopsis* leaves and leaf trichome. **(D,E)** *PAtERG2*::GUS fusion staining in flowers and pollen at BP (before pollination) and 0.5 DAP (day after pollination), respectively. **(F–I)** *PAtERG2*::GUS fusion staining in developed seeds at BP, 0.5, 1.0, and 1.5 DAP, respectively. The central polar nuclei are indicated by cn; the egg cells are indicated by ec; the synergid cells are indicated by sc; the central triploid nucleus is indicated by cc; the endosperm are indicated by end; the embryos at zygote are indicated by zy; and the early globe-stage embryos (with fewer than four cells in a single embryo) are indicated by ea gl. **(J)** The transcription level of *AtERG2* is detected by qRT-PCR in different tissues of *Arabidopsis*. UBQ10 was used as internal control. Error bars indicate the SD of three biological replicates. Different letters (a–e) indicate statistically significant differences (*p* < 0.05) according to Tukey's multiple range test.

To detect the transcription pattern of *AtERG2* in *Arabidopsis*, we first generated the independent transgenic *Arabidopsis* lines, which stably expressed *P*_*AtERG*2_*::GUS* (Figure 2). In the F2 generation, GUS activity was observed in the leaf vein of 1-week-old seedling cytoledons (Figure 2A), trichomes of 3-week-old rosette leaves (Figures 2B,C), pollen grains inside the anthers (Figures 2D,E), and ovules during the period of pollination and early-stage seed development (Figures 2F–I). In anthers, GUS activity was relatively weak the day before pollination (Figure 2D), but it increased in mature pollen at day 0.5 after pollination (DAP) (Figure 2E). We also found that GUS staining was detected at a low level in the pre-matured embryo sac before pollination (Figure 2F); it then reached the highest level in the ovule of 0.5- and 1.0-DAP seeds during the zygotic stage (Figures 2G,H) and quickly dropped in the 1.5-DAP seeds as soon as it reached the early-globe stage (Figure 2I). These results were further confirmed by qRT-PCR assays to evaluate the expression of AtERG2 in different tissues and in the development of *Arabidopsis*. Figure 2J showed that AtERG2 transcripts were highly expressed in the 0.5-, 1.0-, and 1.5-DAP seeds.

### The T-DNA insertion lines of *AtERG2* shows a sporophytic maternal effect phenotype

**Figure 3:**
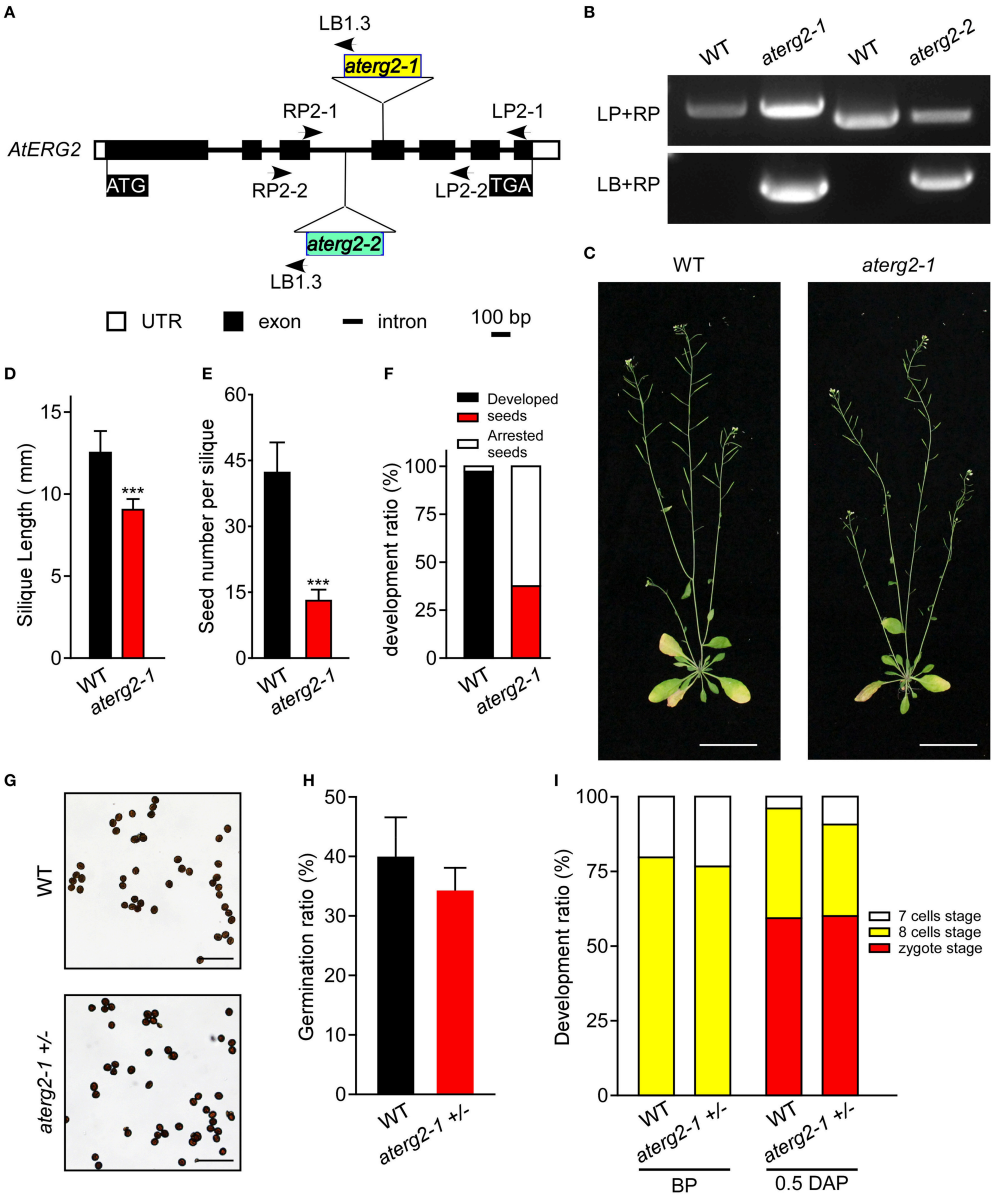
*AtERG2* is essential for early seedling development. **(A)** The schematic diagram for two T-DNA insertions in the *Arabidopsis* genome of *aterg2*. Two lines, SALK_032115 (*aterg2-1*) and SALK_032124 (*aterg2-2*), were purchased from The *Arabidopsis* Information Resource (TAIR, http://www.arabidopsis.org/). Open box, UTR; black box, exon; line, intron. Bar = 100 bp. **(B)** The expression of *aterg2-1* and *aterg2-2* identified by PCR. *Arabidopsis* wild-type (WT) was used as the control. LP, LB, and RP primers were the primers used for PCR and are shown in Table S1. **(C)** The 6-week-old whole aerial parts of the WT and heterozygous line *aterg2-1* +*/*− after planting in soil. Bar = 5 cm. **(D)** The length of mature siliques from WT and *aterg2-1* +*/*− (*n* = 60). The values are means ± SD. ^***^ indicates extremely statistically significant values (*P* < 0.001). **(E)** The mature seed numbers per silique of WT, *aterg2-1*, and *aterg2-2* plants (*n* = 60). The values are means ± SD. ^***^ indicates high levels of statistical significance (*P* < 0.001). **(F)** The ratio of developed and arrested seeds in WT, *aterg2-1*, and *aterg2-2* plants at 6 DAP (number of siliques = 8). **(G)** The iodine-staining of mature pollens from WT and *aterg2-1* +*/*−. Bar = 0.2 mm. **(H)** The germination ratio of mature pollens from WT and *aterg2-1* +*/*−. The pollen number >260. **(I)** The ratio of developed ovules in WT and *aterg2-1* +*/*− at BP (before pollination) and 0.5-DAP, respectively. The ovule number >150.

To characterize the function of AtERG2, the two T-DNA insertion lines (SALK_032115, named as *aterg2-1*, and SALK_032124, as *aterg2-2*, respectively, Figure 3A) were obtained from The *Arabidopsis* Information Resource (TAIR, https://www.arabidopsis.org/), and the genotype of the progeny from self-crossed plants was examined by PCR. In 25 randomly selected mature siliques from self-crossed *aterg2-1* +*/*− plants, we found 687 aborted and 319 available seeds, in which 153 seeds are WT and 166 seeds are hemizygous (Figure 3B, Table 1). These results suggest that the insertion inactivation of AtERG2 severely affect the seed development. During the whole growth period from germination to flowering, *aterg2-1*+*/*− showed similar phenotypes to WT, with the exception of silique length and the mature seed number, both of which were significantly lower in the hemizygous lines compared to the WT (Figures 3C–E). Because *aterg2-1* +*/*− and *aterg2-2* +*/*− had similar phenotypes, we selected the data on *aterg2-1* +*/*− for presentation; the subsequent analyses were performed using *aterg2-1* +*/*− as the study material. Moreover, the developed seeds per silique in *aterg2-1*+*/*− were about 30% of WT at 6.0 DAP until seed maturation (Figure 3F). These results indicate that seed development was arrested and aborted at the early development of seeds in *aterg2-1* +*/*−.

**Table 1:** The genotype of F1 progeny of *aterg2* +*/*− self-crossed or back-crossed with WT.

**Parental Genotype (Female x Male)^a^**	**Available seeds**		**aborted seeds**
	***AtERG2***	***aterg2* +/−**	
*aterg2* +/− × *aterg2* +/−	153	166	687
*aterg2* +/− × *AtERG2*	21	10	N/A
*AtERG2* × *aterg2* +/−	47	36	N/A

a *25 mature siliques were taken randomly from self-crossed aterg2 +/− plants; 6 siliques from back-crossed aterg2 +/− with WT when using WT as a paternal provider; 5 siliques from back-crossed aterg2 +/− with WT when using aterg2 +/− as a paternal provider. The genotypes of the progeny were determined by PCR*.

In order to explore which development stage was abnormal during the reproductive period in *aterg2-1* +*/*− plant, we first backcrossed *aterg2-1* +*/*− with WT plants and found that 21 WT and 10 hemizygous available seeds in 6 backcrossed siliques when using WT as a paternal provider, and 47 WT and 36 hemizygous seeds in 5 backcrossed siliques when using WT as the maternal provider (Table 1). In addition, staining of the mature pollens with iodine and measurement of the *in vitro* germination of pollens revealed no significant differences in the starch accumulation or the pollen germination ratio of WT and *aterg2-1* +*/*− (Figures 3G,H). Similarly, we did not detect any significant difference in the development of embryo sacs between WT and *aterg2-1* +*/*− plants at the pre-pollination stage or at 0.5 DAP (Figure 3I and Figure S3). Therefore, our results suggest that *aterg2-1* +*/*− exhibited a sporophytic maternal effect (SME) phenotype, suggesting that AtERG2 plays a vital role in the early stage of seed development in *Arabidopsis*, but it does not affect gametophyte development.

### *Aterg2-1* +/− shows defects in early embryo development

**Figure 4:**
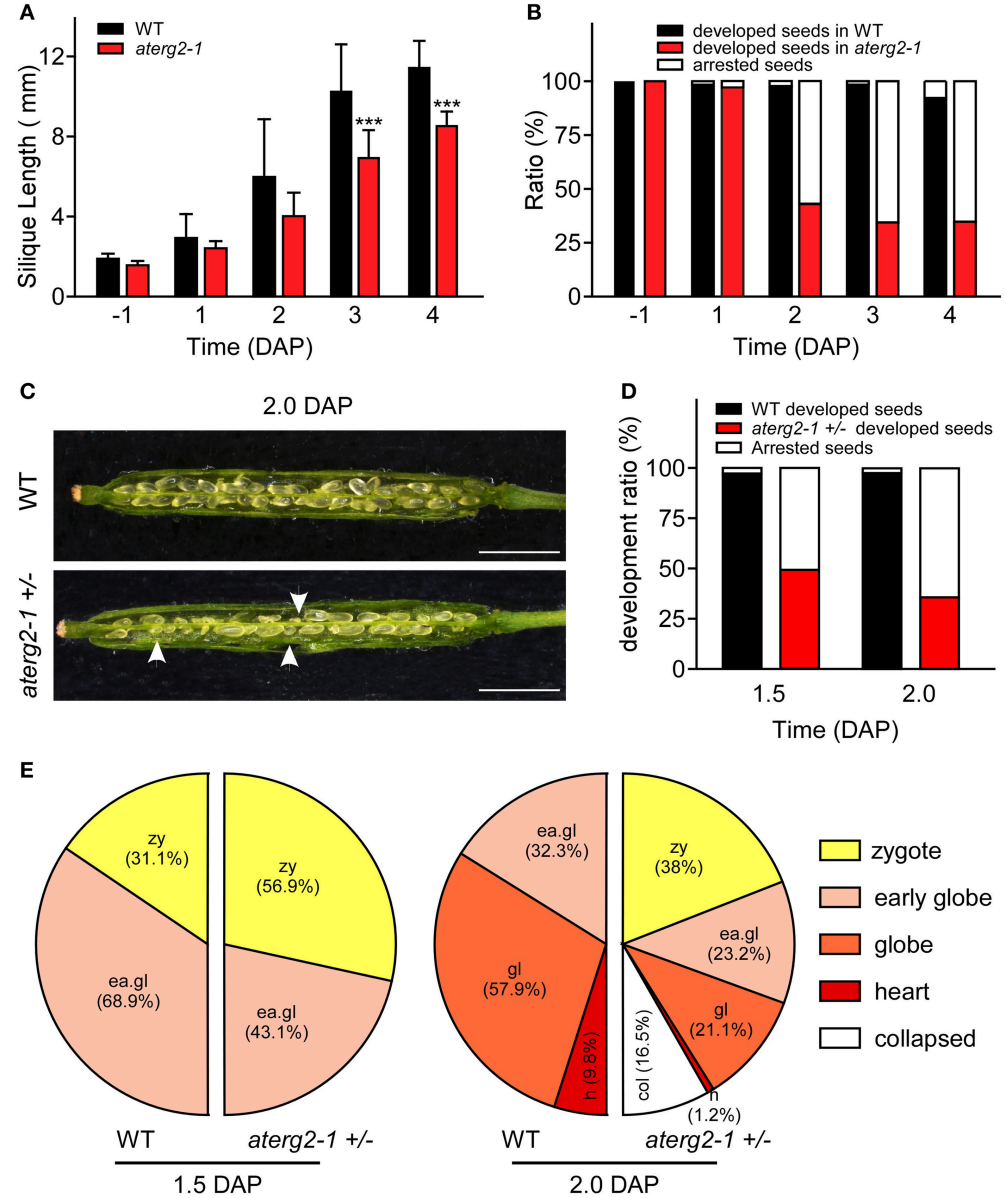
The development phenotypes of early seeds and ovules in WT and *aterg2-1* +*/*−. **(A)** The silique length of WT and *aterg2-1* +*/*− measured from the day before pollination to four DAP (*n* = 16). The values are means ± SD. ^***^ indicates extremely statistically significant values (*P* < 0.001). **(B)** The seed abortion ratio of WT and *aterg2-1* +*/*− calculated from the day before pollination to 4 DAP (silique number = 8). **(C)** The opened 2.0-DAP siliques from WT (top) and *aterg2-1* +*/*− (bottom). The arrows point to the aborted seeds. Bar = 1 mm. **(D)** The seed abortion ratio of WT and *aterg2-1* +*/*− at 1.5- and 2.0-DAP, respectively. Silique numbers = 8. **(E)** The ratio of developed ovules in WT and *aterg2-1* +*/*− at 1.5- and 2.0-DAP, respectively. The seed numbers of WT and *aterg2-1* +*/*− at 1.5-DAP are 190 and 167, respectively; that of WT and *aterg2-1* +*/*− at 2.0-DAP are 183 and 237, respectively.

We investigated the silique development of WT and *aterg2-1* +*/*− from the pre-pollination stage (BP) to 4.0 DAP and found that the silique length was significantly shorter from 3.0 DAP in *aterg2-1* +*/*− than it was in WT (Figure 4A). Some seeds were small and arrested at 1.0 DAP, and the arrested ratio of seed development reached 65% at 3.0 DAP in *aterg2-1* +*/*− (Figure 4B), which is almost the same as the ratio of seed development at 6.0 DAP (Figure 3F). Additionally, we dissected the siliques and hyalinized seeds to observe the embryo development at 1.5 and 2.0 DAP to explore the impairment of early seed development in *aterg2-1* +*/*−. There are 50.7 and 64.2% small-sized seeds at 1.5 DAP and 2.0 DAP in *aterg2-1* +*/*− silique, respectively, but only 2.4% were found in WT silique; however, the average silique length was the same in WT and *aterg2-1* +*/*− plants at 2.0 DAP (Figures 4C,D). At 1.5 DAP, there were two types of embryos in WT and *aterg2-1* +*/*− siliques: 31.1% in the zygote stage and 68.9% in the early-globe stage for WT and 56.9% in the zygote stage and 43.1% in the early globe-stage embryos for *aterg2-1* +*/*− siliques, respectively (Figure 4E and Figure S3). Furthermore, we found 9.8% heart-stage, 57.9% globe-stage (eight or more cells of embryos), and 32.2% early globe-stage embryos in 2.0-DAP WT seeds, and 1.2% heat-stage, 21.1% globe-stage, and 23.3% early globe-stage embryos were found in 2.0-DAP *aterg2-1* +*/*− seeds. More interestingly, as much as 38% of seeds stayed in the zygote stage, and 16.5% of these seeds were collapsed seeds without embryos in 2.0-DAP *aterg2-1* +*/*− siliques (Figure 4E and Figure S3). These results suggested that insertional inactivation of AtERG2 causes delay in the development of embryos after pollination and arrested seeds from 1.5 DAP in *aterg2-1* +*/*− plants.

### The *aterg2-1* +/− ovules at 1.5 and 2.0 DAP show unusual tissue degradation inside the embryo sacs

**Figure 5:**
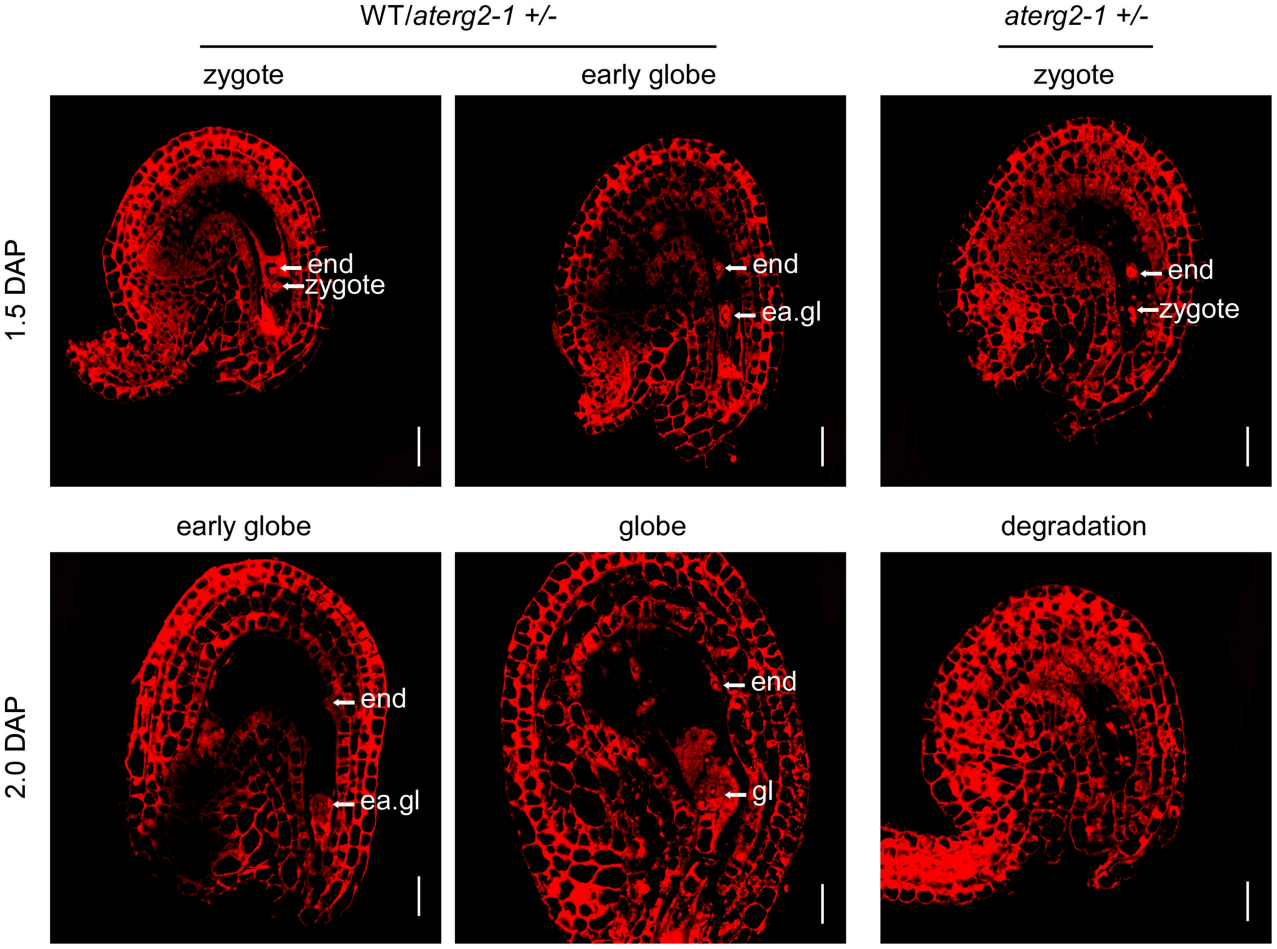
The ovule structure of 1.5- and 2.0-DAP seeds from WT and *aterg2-1* +*/*−. Whole-mount Eosin B staining-confocal laser scanning microscopy (WE-CLSM) analysis of 1.5- and 2.0-DAP seeds of WT and *aterg2-1* +*/*−. The endosperm are indicated by end; the embryos at the zygote stage are indicated by zy; the early globe-stage embryos (with fewer than four cells in a single embryo) are indicated by ea. gl; globe-stage embryos are indicated by gl. Bar = 20 μm.

We performed WE-CLSM analysis to monitor the development of WT and *aterg2-1*+*/*− seeds at 1.5 and 2.0 DAP. We found that, similar to WT, most *aterg2-1*+*/*− seeds at 1.5 DAP developed into the zygote stage and early globe stage (Figure 5 and Table S2). However, some of the hemizygous seeds in the zygote stage presented with abnormal tissue degradation surrounding the endosperm and embryo (Figure 5 and Table S2). Moreover, a number of heterozygous seeds at 2.0 DAP showed severe tissue degradation, revealing the dissolved content inside the embryo sac and seed coat invagination (Figure 5 and Table S2). These data provide a plausible explanation for the observed collapsed phenotype observed in the *aterg2-1* +*/*− siliques at 2.0 DAP (Figure 4E and Figure S3).

### ROS accumulation, activated transcription of ROS-responsible and cell death-related genes in *aterg2-1* +/− arrested seeds

**Figure 6:**
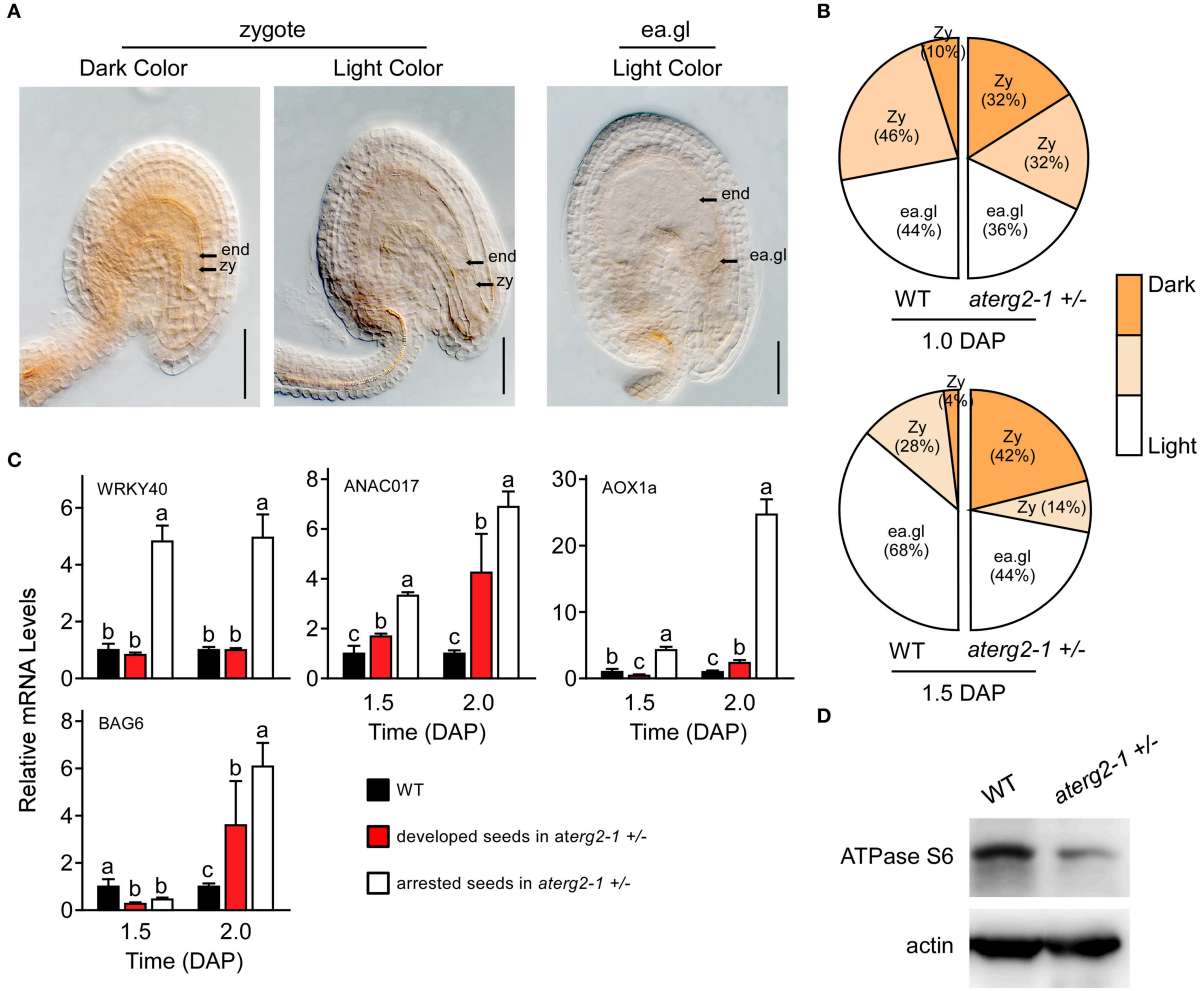
ROS accumulation, and expression of ROS-responsible and cell death-related genes in the arrested seeds of *aterg2-1* +*/*−. **(A)** DAB staining of 1.0- and 1.5-DAP seeds of WT and *aterg2-1* +*/*−. The seeds were observed under 40x objective DIC bright-field microscope. The zygote-stage embryo is indicated by zy; the early globe-stage embryos (with fewer than four cells in a single embryo) are indicated by ea. gl; the endosperm cell is indicated by end. Bar = 0.05 mm. **(B)** Statistical analysis of DAB staining in 1.0- and 1.5-DAP seeds of WT and *aterg2-1*. The zygote-stage embryo is indicated by zy; the early globe-stage embryos (with fewer than four cells in a single embryo) are indicated by ea. gl. Color bar represents the DAB staining from light to dark color. **(C)** The relative expression levels of ROS-responsible genes *WRKY40, ANAC017*, and *AOX1a*, and cell death-related genes *AtBAG6* were determined using qRT-PCR in 1.5- and 2.0-DAP seeds of WT and *aterg2-1*. The mRNA levels were normalized to that of *actin*. The dark bars represent the WT seeds, and the red and white bars represent the developed and arrested seeds of *aterg2-1*, respectively. Error bars indicate the SD of three biological replicates. Different letters (a–c) indicate statistically significant differences (*p* < 0.05) according to Tukey's multiple range test. **(D)** Western blot to detect the protein level of mitochondrial synthesized protein ATPase Subunit 6 in 1.5-DAP siliques of WT and *aterg2-1* +*/*−. An equal amount of protein (10 μg) was loaded into each lane. Actin is used as the marker of protein loading control.

Previous studies showed that the knockdown of ERAL1 in human cell lines by siRNA inhibits mitochondrial protein translation and elevates mitochondrial ROS production, leading to cell death (Uchiumi et al., 2010; Xie et al., 2012). Thus, ROS production and the expression of the ROS-related genes may cause the developmental stagnation of embryos and arrested seeds in *aterg2-1* +*/*−. To test this hypothesis, we first performed DAB staining to monitor the ROS content in *Arabidopsis* seeds after pollination. We found that about 32% zygote-stage seeds in *aterg2-1* +*/*− at 1.0 DAP exhibited more pronounced increases in DAB staining (dark color), but only 10% zygote-stage seeds in WT showed strong DAB staining (Figures 6A,B). At 1.5 DAP, about 42% of *aterg2-1* +*/*− zygote-stage seeds exhibited strong DAB staining compared to the 4% in WT seeds (Figures 6A,B). At 0.5 and 2.0 DAP however, none of the WT or hemizygous seeds showed dark color DAB staining (data not shown). Thus, these data suggest that the accumulation of ROS occurred prior to the abortion of seed development in *aterg2-1* +*/*− plants.

Furthermore, *Alternative oxidase1a* (*AOX1a*), a nuclear gene encoding mitochondrial protein, is activated by ROS through ROS-responsible transcription factors WRKY40 and ANAC017 (Ng et al., 2013; Van Aken et al., 2013; Liu et al., 2014). Thus, these three genes can act as the marker genes for monitoring ROS levels. qRT-PCR showed that the transcription levels of these three genes was specifically higher at 1.5 and 2.0 DAP in the arrested seeds of *aterg2-1* +*/*− than in the developed seeds of WT and *aterg2-1* +*/*− (Figure 6C). Additionally, BCL2-associated athanogene (BAG) family, as the calmodulin-binding proteins, serve as co-chaperones to recruit HSP70/HSC70 to a specific target protein regulating diverse cellular pathways, such as programmed cell death (PCD) and stress responses in eukaryotes (Hung et al., 2003; Yan et al., 2003). Kang et al. (2006) showed that overexpression of *AtBAG6* induced cell death phenotypes consistent with PCD in yeast and plants. Here, we found that *AtBAG6* is extremely up-regulated in 2.0-DAP seeds of *aterg2-1* +*/*− but not in WT plants (Figure 6B), which suggested that inactivation of *AtERG2* induces the higher ROS levels and the cell death of embryos after pollination to produce the arrested seeds in *aterg2-1* +*/*− plants.

### Decreased protein levels of mitochondria-encoded genes in *aterg2-1* +/− arrested seeds

To confirm whether AtERG2 regulates early seed development via affecting the translation of mitochondrial DNA-encoded genes, we performed western blot assay to detect the protein level of some mitochondria-encoded genes. ATPase subunit 6 (ATPase S6) is encoded by the mitochondrial DNA, transcribed and translated in mitochondria, and assembled into the mitochondrial proton-transporting ATP synthase complex. We collected the 2-DAP (including 1.5- and 2.0-DAP) siliques of WT and *aterg2-1* +*/*− plants and extracted the total proteins to quantify the levels of ATPase S6. Western blot assays showed that the protein level of ATPase S6 in *aterg2-1* +*/*− siliques was significantly lower than that in the WT siliques (Figure 6D). The original images of Figure 6D is shown in Figure S4, suggesting that AtERG2 is essential for mitochondrial protein synthesis and that *aterg2-1* deficiency causes ROS-induced cell death due to mitochondria protein dysfunction.

## Discussion

After double fertilization in flowering plants, seed development starts with the coordinated development of embryo and endosperm, as well as interactions between endosperm and seed coat within maternal sporophytic tissue and requires both parental genomes. Previous studies showed that embryo development is dominantly affected by both the female gametophyte and the sporophytic tissue of the parent plant, which is regulated by two classes of maternal effect genes: the SME genes, which are required in the maternal sporophyte for proper embryo development, and the gametophytic maternal effect (GME) genes, which are required in the female gametophyte for proper embryo development (Grossniklaus et al., 1998; Evans and Kermicle, 2001; Pien and Grossniklaus, 2007). Based on high-throughput sequencing, Autran et al. (2011) showed that maternal epigenetic regulation controls the parental contributions in early embryogenesis in *Arabidopsis*. Additionally, mitochondria act as another maternal inheritance factor that affects the early seed development, although the ultrastructure observations implied that mitochondria could also be transmitted from sperm to the egg cell in tobacco (Yu and Russell, 1994). In this study, we found that AtERG2 is a nucleus-encoded and mitochondria-localized ERA-like GTPase in *Arabidopsis*, and the T-DNA insertion hemizygous plants of *AtERG2* show a SME phenotype after self-crossing or back-crossing with WT, which suggested that *aterg2* +*/*− represents a distinct class of mutants affecting seed development.

The *Arabidopsis* genome contains three homologs of ERG: AtERG1 is localized in the chloroplast, AtERG2 is localized in mitochondria (Suwastika et al., 2014), and AtDER, similar to chloroplast-localized double Era-like GTPase in *Nicotiana benthamiana* (NbDER) (Jeon et al., 2014), is predicted to be localized in the chloroplast. Here, we further showed that AtERG2 is localized to the mitochondria, dependent on its N-terminal sequence and is mainly expressed in the leaf vein, trichome, mature pollen, and ovule. The heterozygous *aterg2* +*/*− plant showed a severe abortion of seeds in siliques from 1.5 DAP, and more than 50% were arrested in an early stage of development, but only 2.4% were arrested in WT silique. After an additional 8 h of development, 16.5% of the seeds were severely crushed into a group of dead cell aggregates, and only 45.5% of embryos could successfully split out suspensors and develop into the globe stage. Using WE-CLSM analysis, we found that some of the *aterg2-1* +*/*− ovules at 1.5 and 2.0 DAP were arrested at the zygote stage and that they showed abnormal tissue degradation within the embryo sacs. However, almost 100% of embryos developed into globe-stage embryos in WT silique. This phenomenon is similar to ERG function in *Antirrhinum majus* (Ingram et al., 1998), suggesting that mitochondria-localized ERG are essential for early seed development in plants.

The mitochondria genome size ranges from <6 kilobase pairs (kbp) in *Plasmodium falciparum* (the human malaria parasite) to >200 kbp in land plants (Gray et al., 1999), which encode a limited number of mitochondria tRNAs, ribosomal proteins, essential subunits of the different respiratory chain complexes, and some components involved in cytochrome-c biogenesis (Marienfeld et al., 1999). Therefore, mitochondria play a pivotal role in regulating cellular energy homeostasis and redox balance, and the disabled respiratory chain may cause broken electron transport chain balance and result in ROS accumulation in the matrix, possibly leading to cell death (Blackstone and Green, 1999). In this study, we showed that AtERG2 binds mitochondrial ribosome small subunit component 18S rRNA, which may benefit the maturation of 18S rRNA and the assembly of the mitochondrial ribosome. We also demonstrated that the protein level of ATPase S6, which is encoded by the mitochondrial gene, *ATMG00410*, and translated in mitochondria, was lower in the 2-DAP siliques of *aterg2-1* +*/*− than it was in those of WT. Additionally, using DAB staining, we found that the ROS level was higher in a number of zygote-stage 1.0- and 1.5-DAP seeds in *aterg2-1* +*/*− plants compared to those in WT. These results indicate that disruption of AtERG2 affected the translation of the respiratory chain-related proteins to trigger ROS accumulation in *aterg2-1* +*/*− hemizygous seeds at the early stage of development. Furthermore, the expression of the ROS-related genes WRKY40, ANAC017, and AOX1a and the cell death-related gene BAG6 was upregulated, suggesting some of the embryos at the zygote stage were arrested and aborted in the mature silique in *aterg2-1* +*/*− hemizygous plants. These results are in accordance with previous studies, which showed that nuclear- or mitochondria-encoded gene mutations affect mitochondrial functions, such as transcription, protein synthesis, and oxidative phosphorylation (Maruyama et al., 2016; Pratibha et al., 2017). In these studies, these mutations altered ROS levels in developing gametophytes, thereby adversely affecting gametophyte and polar nuclei fusion during seed development (Maruyama et al., 2016; Pratibha et al., 2017). In our study however, mutation in AtERG2 affected ROS levels in the embryo sac following double fertilization, which was associated with degradation of the embryo sac contents and collapse of the seed coat, leading to seed abortion. These results highlight the diverse function of the mitochondria during seed development in plants.

## Accession numbers

Sequence data for the genes in this study can be found in the GenBank database under the following accession numbers: AtERG2 (At1g30970), ANAC017 (AT1G34190), AOX1a (AT3G22370), WRKY40 (AT1G80840), BAG6 (AT2G46240), mitochondrial ribosome large subunit component 26S rRNA (AtMG00020), mitochondrial ribosome small subunit 18S rRNA (AtMG01390), and mitochondrial ATPase Subunit 6 (ATMG00410).

## Author contributions

Conceived and designed the experiments: SH, JZ, HxZ, HpZ, and PC. Performed the experiments: PC, HjL, LY, HyL, LX, and JL. Analyzed the data: PC, HjL, YW, HpZ, HxZ, and SH. Contributed reagents, materials, analysis tools: HpZ, HxZ, and SH. Wrote the paper: PC, HpZ, HxZ, and SH.

### Conflict of interest statement

The authors declare that the research was conducted in the absence of any commercial or financial relationships that could be construed as a potential conflict of interest.

## Supplementary material

Figure S1 Phylogenetic analysis of AtERGs and homologous proteins. Phylogenetic tree constructed using the predicted amino acid sequences of the 21 ERA homologous proteins in 14 species. ERA (NP_417061.1) and EcDER (double Era-like GTPase, AAC75564) were from *E. coli*; ERAL1 (NP_005693.1) from *Homo sapiens*; DrERA (NP_001122219.1) from *Danio rerio*; MmERA (NP_071708.2) from *Mus musculus*; ScERA (NP_013736.1) from *Saccharomyces cerevisiae*; ERG (O82626.1) from *Antirrhinum majus*; AtERG1 (At5g66470), AtERG2 (At1g30960), and AtDER (At3g12080) from *Arabidopsis thaliana*; OsERG1 (Os05g49220), OsERG2 (Os08g10649.1), and OsDER (XP_015628913.1) from *Oryza sativa*; ZmERG (NP_001167959.1) and ZmDER (ACL53683) from *Zea mays*; StERG (XP_006363190.1) from *Solanum tuberosum*; CsERG (XP_004134545.1) from *Cucumis sativus*; NtERG (XP_016481550.1) from *Nicotiana tabacum*; SlERG (XP_004232641.1) from *Solanum lycopersicum*; NbDER (KC846070) from *Nicotiana benthamiana*.

Figure S2 Schematic of different AtERG2-eYFP constructs. The full-length and different truncated AtERG2 constructs were constructed with eYFP (green rectangles) attached to the C-terminal region. AtERG2 was divided into three different regions: 1–150 aa as the N-terminal of AtERG2 (AtERG2_N); 151–437 aa as the C-part of AtERG2 (AtERG2_C), which contains 151–326 aa as GTPase domain (AtERG2_GD); and 338–437 aa as the KH-domain (AtERG2_KH).

Figure S3 The ovule development in WT and *aterg2-1* +*/*− from before pollination (BP) to 2.0 DAP. The seeds were observed under a 40x objective DIC bright field microscope. “ec” = egg cells; “sy” = synergid cells; “pc” = the two polar nuclei; “cc” = central cells following the fusion of polar cells; “zy” = embryos in the zygote stage; “ea. gl” = embryos in the early globe stage (containing <4 cells in a single embryo); “end” = endosperm cells; “gl” = embryos in the globe stage; “h” = embryos in the heart stage. Bar = 0.1 mm.

Figure S4 The original image of Figure 6D. Western blot to detect the protein level of mitochondrial synthesized protein ATPase Subunit 6 in 1.5-DAP siliques of WT and *aterg2-1* +*/*−. An equal amount of protein (10 μg) was loaded into each lane. Actin is used as the marker of the cytoplasm component.

Table S1 Primer sequences used in this study.

Table S2 WE-CLSM analysis of embryo development in WT and *aterg2-1* +*/*− at 1.5 and 2.0 DAP.

## References

[B1] Ahnn J. March P. E. Takiff H. E. Inouye M. (1986). A GTP-binding protein of *Escherichia coli* has homology to yeast RAS proteins. Proc. Natl. Acad. Sci. U.S.A. 83, 8849–8853. 10.1073/pnas.83.23.8849 3097637 PMC387030

[B2] Autran D. Baroux C. Raissig M. T. Lenormand T. Wittig M. Grob S.. (2011). Maternal epigenetic pathways control parental contributions to arabidopsis early embryogenesis. Cell 145, 707–719. 10.1016/j.cell.2011.04.014 21620136

[B3] Blackstone N. W. Green D. R. (1999). The evolution of a mechanism of cell suicide. Bioessays 21, 84–88. 10.1002/(SICI)1521-1878(199901)21:1<84::AID-BIES11>3.3.CO;2-S 10070258

[B4] Britton R. A. Chen S. M. Wallis D. Koeuth T. Powell B. S. Shaffer L. G.. (2000). Isolation and preliminary characterization of the human and mouse homologues of the bacterial cell cycle gene era. Genomics 67, 78–82. 10.1006/geno.2000.6243 10945472

[B5] Britton R. A. Powell B. S. Court D. L. Lupski J. R. (1997). Characterization of mutations affecting the Escherichia coli essential GTPase era that suppress two temperature-sensitive dnaG alleles. J. Bacteriol. 179, 4575–4582. 10.1128/jb.179.14.4575-4582.1997 9226268 PMC179294

[B6] Clough S. J. Bent A. F. (1998). Floral dip: a simplified method for Agrobacterium-mediated transformation of *Arabidopsis thaliana*. Plant J. 16, 735–743. 10.1046/j.1365-313x.1998.00343.x 10069079

[B7] Daudi A. Cheng Z. O'Brien J. A. Mammarella N. Khan S. Ausubel F. M.. (2012). The apoplastic oxidative burst peroxidase in Arabidopsis is a major component of pattern-triggered immunity. Plant Cell 24, 275–287. 10.1105/tpc.111.093039 22247251 PMC3289579

[B8] Dennerlein S. Rozanska A. Wydro M. Chrzanowska-Lightowlers Z. M. Lightowlers R. N. (2010). Human ERAL1 is a mitochondrial RNA chaperone involved in the assembly of the 28S small mitochondrial ribosomal subunit. Biochem. J. 430, 551–558. 10.1042/bj20100757 20604745 PMC2995420

[B9] Edwards K. Johnstone C. Thompson C. (1991). A simple and rapid method for the preparation of plant genomic DNA for PCR analysis. Nucleic Acids Res. 19:1349. 10.1093/nar/19.6.1349 2030957 PMC333874

[B10] Evans M. M. Kermicle J. L. (2001). Interaction between maternal effect and zygotic effect mutations during maize seed development. Genetics 159, 303–315. 11560906 10.1093/genetics/159.1.303 PMC1461788

[B11] Gray M. W. Burger G. Lang B. F. (1999). Mitochondrial evolution. Science 283, 1476–1481. 10.1126/science.283.5407.1476 10066161

[B12] Grossniklaus U. Vielle-Calzada J. P. Hoeppner M. A. Gagliano W. B. (1998). Maternal control of embryogenesis by medea, a Polycomb group gene in Arabidopsis. Science 280, 446–450. 10.1126/science.280.5362.446 9545225

[B13] Hung W. J. Roberson R. S. Taft J. Wu D. Y. (2003). Human BAG-1 proteins bind to the cellular stress response protein GADD34 and interfere with GADD34 functions. Mol. Cell. Biol. 23, 3477–3486. 10.1128/mcb.23.10.3477-3486.2003 12724406 PMC164759

[B14] Inada T. Kawakami K. Chen S. M. Takiff H. E. Court D. L. Nakamura Y. (1989). Temperature-sensitive lethal mutant of era, a G protein in *Escherichia coli*. J. Bacteriol. 171, 5017–5024. 10.1128/jb.171.9.5017-5024.1989 2527846 PMC210312

[B15] Ingram G. C. Simon R. Carpenter R. Coen E. S. (1998). The Antirrhinum ERG gene encodes a protein related to bacterial small GTPases and is required for embryonic viability. Curr. Biol. 8, 1079–1082. 10.1016/s0960-9822(98)70445-2 9768362

[B16] Jeon Y. Ahn C. S. Jung H. J. Kang H. Park G. T. Choi Y.. (2014). DER containing two consecutive GTP-binding domains plays an essential role in chloroplast ribosomal RNA processing and ribosome biogenesis in higher plants. J. Exp. Bot. 65, 117–130. 10.1093/jxb/ert360 24272962 PMC3883289

[B17] Kang C. H. Jung W. Y. Kang Y. H. Kim J. Y. Kim D. G. Jeong J. C.. (2006). AtBAG6, a novel calmodulin-binding protein, induces programmed cell death in yeast and plants. Cell Death Differ. 13, 84–95. 10.1038/sj.cdd.4401712 16003391

[B18] Leipe D. D. Wolf Y. I. Koonin E. V. Aravind L. (2002). Classification and evolution of P-loop GTPases and related ATPases1. J. Mol. Biol. 317, 41–72. 10.1006/jmbi.2001.5378 11916378

[B19] Lerner C. G. Inouye M. (1991). Pleiotropic changes resulting from depletion of Era, an essential GTP-binding protein in *Escherichia coli*. Mol. Microbiol. 5, 951–957. 10.1111/j.1365-2958.1991.tb00770.x 1906969

[B20] Liang M. Li H. Zhou F. Li H. Liu J. Hao Y.. (2015). Subcellular distribution of NTL transcription factors in *Arabidopsis thaliana*. Traffic 16, 1062–1074. 10.1111/tra.12311 26201836

[B21] Liu C. M. Meinke D. W. (1998). The titan mutants of Arabidopsis are disrupted in mitosis and cell cycle control during seed development. Plant J. 16, 21–31. 10.1046/j.1365-313x.1998.00268.x 9807824

[B22] Liu J. Li Z. Wang Y. Xing D. (2014). Overexpression of ALTERNATIVE OXIDASE1a alleviates mitochondria-dependent programmed cell death induced by aluminium phytotoxicity in Arabidopsis. J. Exp. Bot. 65, 4465–4478. 10.1093/jxb/eru222 24863436

[B23] Marienfeld J. Unseld M. Brennicke A. (1999). The mitochondrial genome of Arabidopsis is composed of both native and immigrant information. Trends Plant Sci. 4, 1360–1385. 10.1016/S1360-1385(99)01502-2 10562735

[B24] Maruyama D. Ohtsu M. Higashiyama T. (2016). Cell fusion and nuclear fusion in plants. Semin. Cell Dev. Biol. 60, 127–135. 10.1016/j.semcdb.2016.07.024 27473789

[B25] Meier T. I. Peery R. B. Jaskunas S. R. Zhao G. S. (1999). 16S rRNA is bound to era of *Streptococcus pneumoniae*. J. Bacteriol. 181, 5242–5249. 10464193 10.1128/jb.181.17.5242-5249.1999 PMC94028

[B26] Ng S. Ivanova A. Duncan O. Law S. R. Van Aken O. De Clercq I.. (2013). A membrane-bound NAC transcription factor, ANAC017, mediates mitochondrial retrograde signaling in Arabidopsis. Plant Cell 25, 3450–3471. 10.1105/tpc.113.113985 24045017 PMC3809543

[B27] Pien S. Grossniklaus U. (2007). Polycomb group and trithorax group proteins in Arabidopsis. Biochimic. Biophys. Acta 1769, 375–382. 10.1016/j.bbaexp.2007.01.010 17363079

[B28] Pratibha P. Singh S. K. Srinivasan R. Bhat S. R. Sreenivasulu Y. (2017). Gametophyte development needs mitochondrial coproporphyrinogen III oxidase function. Plant Physiol. 174, 258–275. 10.1104/pp.16.01482 28270625 PMC5411134

[B29] Shan S. O. (2016). ATPase and GTPase tangos drive intracellular protein transport. Trends Biochem. Sci. 41, 1050–1060. 10.1016/j.tibs.2016.08.012 27658684 PMC5627767

[B30] Suwastika I. N. Denawa M. Yomogihara S. Im C. H. Bang W. Y. Ohniwa R. L.. (2014). Evidence for lateral gene transfer (LGT) in the evolution of eubacteria-derived small GTPases in plant organelles. Front. Plant Sci. 5:678. 10.3389/fpls.2014.00678 25566271 PMC4263083

[B31] Takai Y. Sasaki T. Matozaki T. (2001). Small GTP-binding proteins. Physiol. Rev. 81, 153–208. 10.1152/physrev.2001.81.1.153 11152757

[B32] Tang Q. Y. Zhang C. X. (2013). Data processing system (DPS) software with experimental design, statistical analysis and data mining developed for use in entomological research. Insect Sci. 20, 254–260. 10.1111/j.1744-7917.2012.01519.x 23955865

[B33] Tu C. Zhou X. Tarasov S. G. Tropea J. E. Austin B. P. Waugh D. S.. (2011). The Era GTPase recognizes the GAUCACCUCC sequence and binds helix 45 near the 3′ end of 16S rRNA. Proc. Natl. Acad. Sci. U.S.A. 108, 10156–10161. 10.1073/pnas.1017679108 21646538 PMC3121871

[B34] Tu C. Zhou X. Tropea J. E. Austin B. P. Waugh D. S. Court D. L.. (2009). Structure of ERA in complex with the 3′ end of 16S rRNA: implications for ribosome biogenesis. Proc. Natl. Acad. Sci. U.S.A. 106, 14843–14848. 10.1073/pnas.0904032106 19706445 PMC2736428

[B35] Uchiumi T. Kang D. (2012). The role of TFAM-associated proteins in mitochondrial RNA metabolism. Biochimic. Biophys. Acta 1820, 565–570. 10.1016/j.bbagen.2011.08.014 21920408

[B36] Uchiumi T. Ohgaki K. Yagi M. Aoki Y. Sakai A. Matsumoto S.. (2010). ERAL1 is associated with mitochondrial ribosome and elimination of ERAL1 leads to mitochondrial dysfunction and growth retardation. Nucleic Acids Res. 38, 5554–5568. 10.1093/nar/gkq305 20430825 PMC2938226

[B37] Van Aken O. Zhang B. Law S. Narsai R. Whelan J. (2013). AtWRKY40 and AtWRKY63 Modulate the expression of stress-responsive nuclear genes encoding mitochondrial and chloroplast proteins. Plant Physiol. 162, 254–271. 10.1104/pp.113.215996 23509177 PMC3641207

[B38] Vernoud V. Horton A. C. Yang Z. Nielsen E. (2003). Analysis of the small GTPase gene superfamily of Arabidopsis. Plant Physiol. 131, 1191–1208. 10.1104/pp.013052 12644670 PMC166880

[B39] Voshol G. P. Meyer V. Van den Hondel C. A. (2015). GTP-binding protein Era: a novel gene target for biofuel production. BMC Biotechnol. 15:21. 10.1186/s12896-015-0132-1 25887126 PMC4380250

[B40] Xie X. Le L. Fan Y. Lv L. Zhang J. (2012). Autophagy is induced through the ROS-TP53-DRAM1 pathway in response to mitochondrial protein synthesis inhibition. Autophagy 8, 1071–1084. 10.4161/auto.20250 22576012 PMC3429544

[B41] Yan J. He C. Zhang H. (2003). The BAG-family proteins in *Arabidopsis thaliana*. Plant Sci. 165, 1–7. 10.1016/S0168-9452(03)00121-3

[B42] Yu H. J. Hogan P. Sundaresan V. (2005). Analysis of the female gametophyte transcriptome of Arabidopsis by comparative expression profiling. Plant Physiol. 139, 1853–1869. 10.1104/pp.105.067314 16299181 PMC1310564

[B43] Yu H. S. Russell S. D. (1994). Occurrence of mitochondria in the nuclei of tobacco sperm cells. Plant Cell 6, 1477–1484. 10.1105/tpc.6.10.1477 12244225 PMC160535

