# Supplementary figures and images for: The ERA-Related GTPase AtERG2 Associated with Mitochondria 18S RNA Is Essential for Early Embryo Development in Arabidopsis
Source: Front Plant Sci. 2018 Feb 15;9:182. doi: 10.3389/fpls.2018.00182 (PMC5818394; doi:10.3389/fpls.2018.00182)

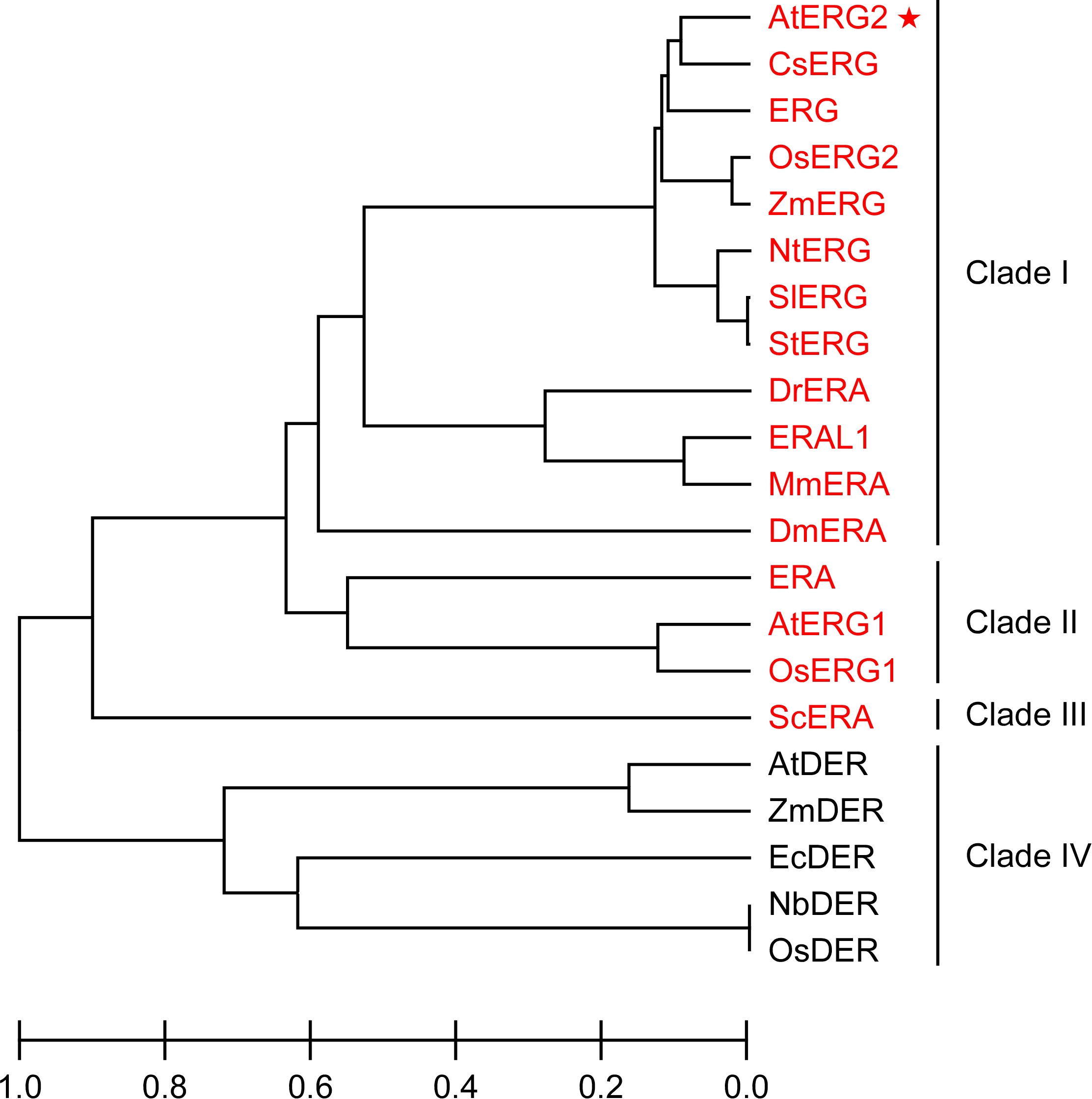

Supplement: Figure S1 — Phylogenetic analysis of AtERGs and homologous proteins. Phylogenetic tree constructed using the predicted amino acid sequences of the 21 ERA homologous proteins in 14 species. ERA (NP_417061.1) and EcDER (double Era-like GTPase, AAC75564) were from E. coli; ERAL1 (NP_005693.1) from Homo sapiens; DrERA (NP_001122219.1) from Danio rerio; MmERA (NP_071708.2) from Mus musculus; ScERA (NP_013736.1) from Saccharomyces cerevisiae; ERG (O82626.1) from Antirrhinum majus; AtERG1 (At5g66470), AtERG2 (At1g30960), and AtDER (At3g12080) from Arabidopsis thaliana; OsERG1 (Os05g49220), OsERG2 (Os08g10649.1), and OsDER (XP_015628913.1) from Oryza sativa; ZmERG (NP_001167959.1) and ZmDER (ACL53683) from Zea mays; StERG (XP_006363190.1) from Solanum tuberosum; CsERG (XP_004134545.1) from Cucumis sativus; NtERG (XP_016481550.1) from Nicotiana tabacum; SlERG (XP_004232641.1) from Solanum lycopersicum; NbDER (KC846070) from Nicotiana benthamiana. [file Image1.JPEG]

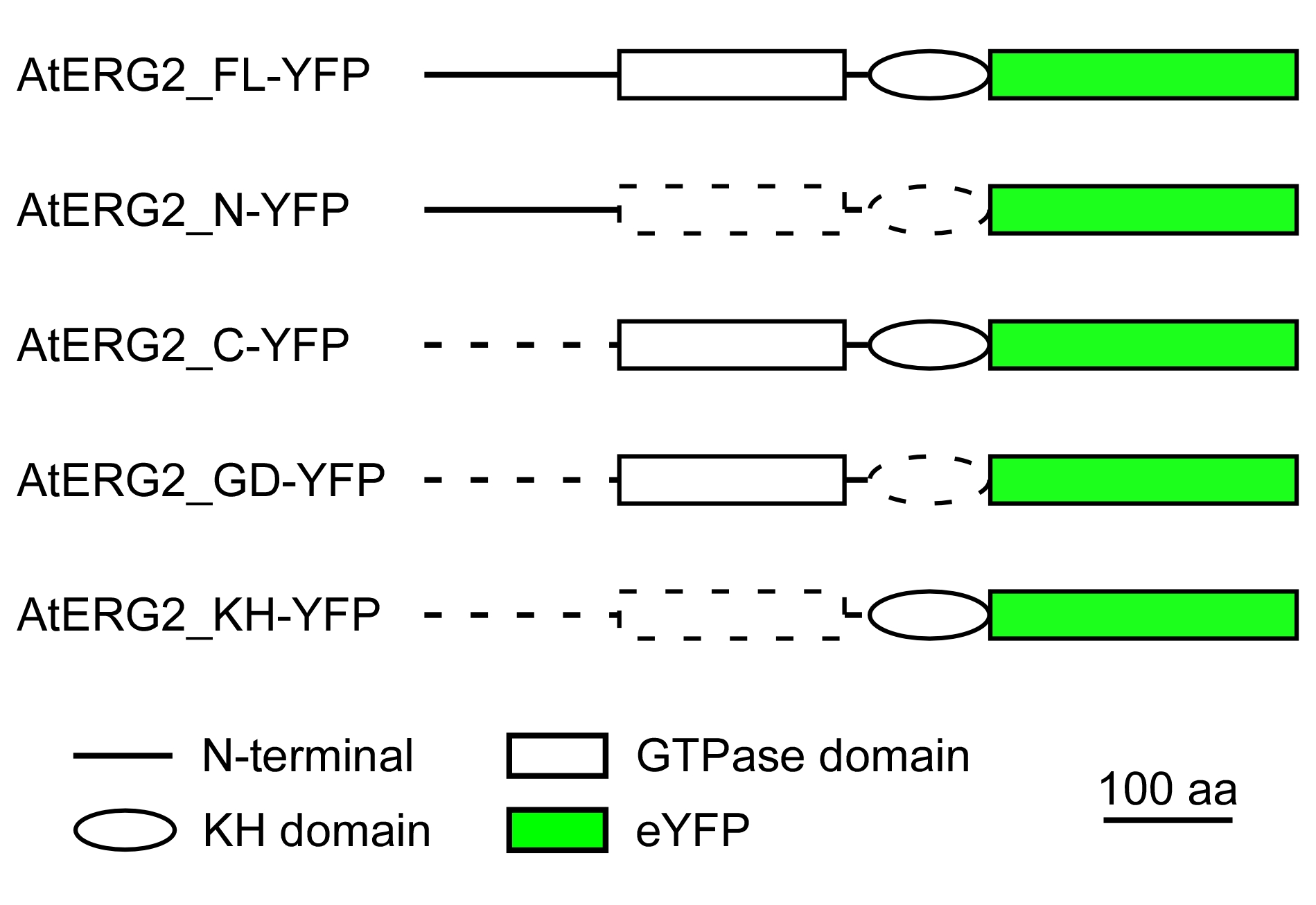

Supplement: Figure S2 — Schematic of different AtERG2-eYFP constructs. The full-length and different truncated AtERG2 constructs were constructed with eYFP (green rectangles) attached to the C-terminal region. AtERG2 was divided into three different regions: 1–150 aa as the N-terminal of AtERG2 (AtERG2_N); 151–437 aa as the C-part of AtERG2 (AtERG2_C), which contains 151–326 aa as GTPase domain (AtERG2_GD); and 338–437 aa as the KH-domain (AtERG2_KH). [file Image2.JPEG]

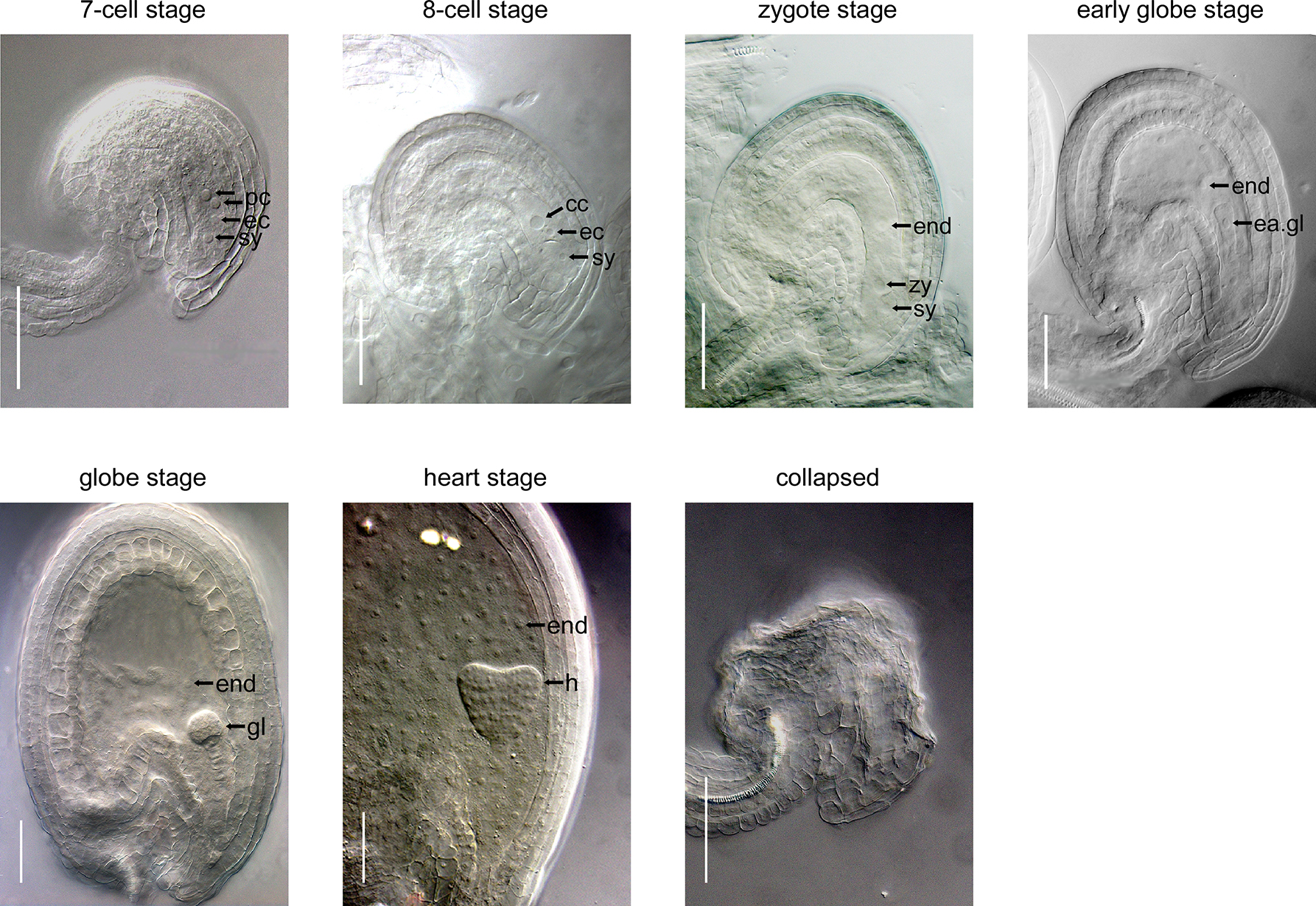

Supplement: Figure S3 — The ovule development in WT and aterg2-1 +/− from before pollination (BP) to 2.0 DAP. The seeds were observed under a 40x objective DIC bright field microscope. “ec” = egg cells; “sy” = synergid cells; “pc” = the two polar nuclei; “cc” = central cells following the fusion of polar cells; “zy” = embryos in the zygote stage; “ea. gl” = embryos in the early globe stage (containing <4 cells in a single embryo); “end” = endosperm cells; “gl” = embryos in the globe stage; “h” = embryos in the heart stage. Bar = 0.1 mm. [file Image3.JPEG]

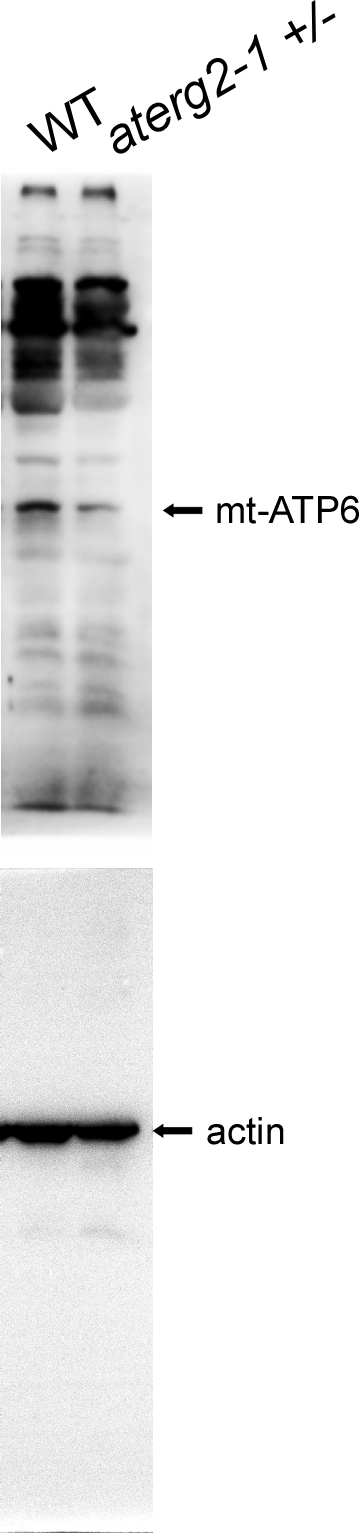

Supplement: Figure S4 — The original image of Figure 6D. Western blot to detect the protein level of mitochondrial synthesized protein ATPase Subunit 6 in 1.5-DAP siliques of WT and aterg2-1 +/−. An equal amount of protein (10 μg) was loaded into each lane. Actin is used as the marker of the cytoplasm component. [file Image4.JPEG]
