# Supplementary material for: The ERA-Related GTPase AtERG2 Associated with Mitochondria 18S RNA Is Essential for Early Embryo Development in Arabidopsis
Source: Front Plant Sci. 2018 Feb 15;9:182. doi: 10.3389/fpls.2018.00182 (PMC5818394; doi:10.3389/fpls.2018.00182)
Supplement: Table S1 — Primer sequences used in this study. [file Table1.DOC]

Table S1 Primer sequences used for this article.

| Purpose | **Name** | **Sequence** |
| --- | --- | --- |
| PCR | | |
| mito 18S rRNA | LP | 5'-AAACCGAAGTGAGCCAAGGA-3’ |
|  | RP | 5'-CGCCAATAAGACCACCAAAA-3’ |
| mito 26S rRNA | LP | 5'-CGTAGTTTGGCGACCTTCAG-3’ |
|  | RP | 5'-CACGACCTCCCACCTATCCT-3’ |
| Vector construction | | |
| AtERG2-N terminal | LP | 5'-GGGGATCCATGAAAGCTTTTAGAT-3' |
|  | RP | 5'-GCGCGGCCGCATCTTCTTCTCTAACTT-3' |
| AtERG2-C terminal | LP | 5'-GGGGATCCATGCAGAAGTCGCTTAATGTC-3' |
|  | RP | 5'-GCGCGGCCGCCTTGAGCTTAACCTG-3' |
| AtERG2-GTPase domain | LP | 5'-GGGGATCCATGCAGAAGTCGCTTAATGTC-3' |
|  | RP | 5'-GCGCGGCCGCATCCATTAAGTATTGGG-3' |
| AtERG2-KH domain | LP | 5'-GGGGATCCATGGTCTTGAAGAACATTT-3' |
|  | RP | 5'-GCGCGGCCGCCTTGAGCTTAACCTG-3' |
| AtERG2-full length | LP | 5'-GGGGATCCATGAAAGCTTTTAGAT-3' |
|  | RP | 5'-GCGCGGCCGCCTTGAGCTTAACCTG-3' |
| Promoter::GUS | | |
| *P_AtERG2_* | LP | 5'-GAAGATCTACCATTTTTGTAACAATGGCG-3' |
|  | RP | 5'-CGGGATCCTTTTGTAACAATGGCGAAGTAGCG-3' |
| qPCR | | |
| ANAC017 | LP | 5'-CCAGCCTCTGCGTGTGAGGG-3' |
|  | RP | 5'-TCACTGGCGTTCCATTAGCCTTTCC-3' |
| AOX1A | LP | 5'-TCCTGCTGATGCGACACT-3' |
|  | RP | 5'-CAAAGAAAGCCGAATCCA-3' |
| WRKY40 | LP | 5'-ACAACCATCCAATGCCATCGCAGA-3' |
|  | RP | 5'-TCTACGGTAGTCACCGGCACAGT-3' |
| BAG6 | LP | 5'-CGTGTTGACCCATTACCG-3' |
|  | RP | 5'-CTGTTTCAGCAGCCGATT-3' |
| BAG4 | LP | 5'-TCGGAATGGGAGGTGAGA-3' |
|  | RP | 5'-AACGGATTTGAACATCTTGC-3' |
| ACTIN | LP | 5'-GGTAACATTGTGCTCAGTGGTGG-3' |
|  | RP | 5'-AACGACCTTAATCTTCATGCTGC-3' |
| AtERG2 | LP | 5'-CTCACGAACGACGACGAAGA-3' |
|  | RP | 5'-ACAGAGTCGCTGCTTGATCC-3' |
| UBQ10 | LP | 5'-GGTGGTTTGTGTTTTGGGGC-3' |
|  | RP | 5'-AGTCGAGTCACTTTGCAGGC-3' |
| T-DNA lines confirmation | | |
| LB1.3 | | 5'-ATTTTGCCGATTTCGGAAC-3’ |
| SALK_032115 | LP2-1 | 5'-TTGGGAGAATGGTTTCACTTG-3’ |
|  | RP2-1 | 5'-GCTTGGACTTCTGTTGACCTG-3’ |
| SALK_032124 | LP2-2 | 5'-CTTGCCTACAAGAATCTTGCG-3' |
|  | RP2-2 | 5'-GTTTCTTCGATACTCCGGGTC-3' |
