# Supplementary material for: The ERA-Related GTPase AtERG2 Associated with Mitochondria 18S RNA Is Essential for Early Embryo Development in Arabidopsis
Source: Front Plant Sci. 2018 Feb 15;9:182. doi: 10.3389/fpls.2018.00182 (PMC5818394; doi:10.3389/fpls.2018.00182)
Supplement: Table S2 — WE-CLSM analysis of embryo development in WT and aterg2-1 +/− at 1.5 and 2.0 DAP. [file Table2.DOC]

Table S2. WE-CLSM analysis of embryo development in WT and *aterg2-1 +/-* at 1.5 and 2.0 DAP.

| Phenotype  Time | | degradation  zygote  (%) | zygote  (%) | early globe  (%) | globe  (%) | heart  (%) | collapsed  (%) |
| --- | --- | --- | --- | --- | --- | --- | --- |
| 1.5 DAP | WT | 0 | 33.7 | 66.3 | 0 | 0 | 0 |
|  | *aterg2-1 +/-* | 17.8 | 40 | 42.2 | 0 | 0 | 0 |
| 2.0 DAP | WT | 0 | 0 | 35 | 57.3 | 7.7 | 0 |
|  | *aterg2-1 +/-* | 27 | 5.7 | 21.3 | 23 | 2.5 | 20.5 |
| n (WT 1.5 DAP) = 98; n (*aterg2-1 +/-* 1.5 DAP) = 135;  n (WT 2.0 DAP) = 117; n (*aterg2-1 +/-* 2.0 DAP) = 122 | | | | | | | |
